# Paralysie de la branche externe du nerf spinal sur cicatrice cheloide

**DOI:** 10.11604/pamj.2016.23.201.9334

**Published:** 2016-04-16

**Authors:** Samia Frioui, Faycel Khachnaoui

**Affiliations:** 1 Service de Médecine Physique et de Réadaptation Fonctionnelle, CHU Sahloul Sousse, Tunisie

**Keywords:** Nerf spinal, paralysie, EMG, chirurgie, Spinal nerve, paralysis, EMG, surgery

## Image en médecine

La paralysie de la branche externe du Nerf Spinal est très rare. Elle réalise un tableau clinique associant une faiblesse et une morphologie anormale de l’épaule. Il faut y penser devant toute chirurgie même simple de la région cervicale. Nous rapportons le cas d'un patient âgé de 20 ans, qui consultait plusieurs médecins pour des douleurs et une faiblesse progressive de l’épaule gauche apparue quelques jours après une reprise d'une cicatrice opératoire chéloïde compliquant une exérèse d'un lipome cervical de 3cm de diamètre réalisée quelques mois auparavant. L'examen clinique retrouvait une force musculaire de l’épaule gauche cotée à 3 sans aucune limitation articulaire, une amyotrophie nette du muscle trapèze homolatéral avec une asymétrie et chute de l’épaule gauche. Devant les éléments de l'examen clinique, une atteinte du Nerf Spinal a été suspectée et un EMG a été demandé. L'EMG objectivait des signes de dénervation totale avec dégénérescence axonale du chef supérieur du trapèze gauche et minime des chefs moyen et inférieur, cadrant avec une lésion partielle du Nerf Spinal gauche. Le patient a été adressé en Chirurgie Plastique et Réparatrice pour réparation nerveuse. L'atteinte de la branche externe du Nerf Spinal se manifeste par des douleurs et une faiblesse de l’épaule déclenchée par les mouvements d'antéflexion du membre supérieur. La cause la plus habituelle est la biopsie ganglionnaire cervicale. Dans notre cas, la lésion du Nerf Spinal est survenue lors de la reprise de la cicatrice cutanée chéloïde. Ceci s'explique par la localisation très superficielle du Nerf Spinal.

**Figure 1:**
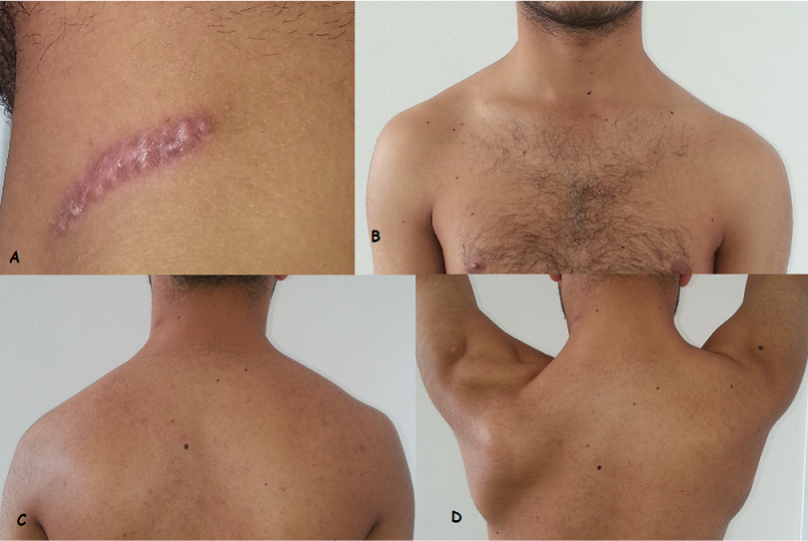
(A) cicatrice au niveau du cou à l'origine de la paralysie de la branche externe du nerf spinal gauche; (B) chute de l’épaule gauche vue de face; (C) chute de l’épaule gauche vue de dos; (D) décollement de l'omoplate gauche lors de l'antépulsion de l’épaule

